# Osmotic Processor for Enabling Sensitive and Rapid Biomarker Detection *via* Lateral Flow Assays

**DOI:** 10.3389/fbioe.2022.884271

**Published:** 2022-06-01

**Authors:** Sheng-You Chen, Abe Y. Wu, Ruby Lunde, James J. Lai

**Affiliations:** ^1^ Department of Mechanical Engineering, University of Washington, Seattle, WA, United States; ^2^ Department of Bioengineering, University of Washington, Seattle, WA, United States

**Keywords:** biomarker concentration, osmosis, polymers, lateral flow tests, limit of detection, biospecimen processing, point-of-care diagnostics

## Abstract

Urine is an attractive biospecimen for *in vitro* diagnostics, and urine-based lateral flow assays are low-cost devices suitable for point-of-care testing, particularly in low-resource settings. However, some of the lateral flow assays exhibit limited diagnostic utility because the urinary biomarker concentration is significantly lower than the assay detection limit, which compromises the sensitivity. To address the challenge, we developed an osmotic processor that statically and spontaneously concentrated biomarkers. The specimen in the device interfaces with the aqueous polymer solution via a dialysis membrane. The polymer solution induces an osmotic pressure difference that extracts water from the specimen, while the membrane retains the biomarkers. The evaluation demonstrated that osmosis induced by various water-soluble polymers efficiently extracted water from the specimens, ca. 5–15 ml/h. The osmotic processor concentrated the specimens to improve the lateral flow assays’ detection limits for the model analytes—human chorionic gonadotropin and SARS-CoV-2 nucleocapsid protein. After the treatment via the osmotic processor, the lateral flow assays detected the corresponding biomarkers in the concentrated specimens. The test band intensities of the assays with the concentrated specimens were very similar to the reference assays with 100-fold concentrations. The mass spectrometry analysis estimated the SARS-CoV-2 nucleocapsid protein concentration increased ca. 200-fold after the osmosis. With its simplicity and flexibility, this device demonstrates a great potential to be utilized in conjunction with the existing lateral flow assays for enabling highly sensitive detection of dilute target analytes in urine.

## 1 Introduction

Urine is one of the most used biospecimens next to blood that can be easily collected in large quantities with noninvasive procedures (Hadland and Levy, 2016). Urine is routinely used at the point-of-care and in laboratory settings to detect pregnancies, diagnose diseases, and screen potential health problems (Tuuminen, 2012). Molecules in urine originate from glomerular filtration of plasma, renal tubule excretion, and shedding of various cells, representing a biomarker repertoire that can be exploited for diagnosis and monitoring of renal as well as systemic diseases (Harpole et al., 2016). Urine is composed of mostly water and solutes like urea, small ions, creatinine, albumin, bilirubin, and low concentrations of other small proteins (Simerville et al., 2005; Taylor and Curhan, 2006). Solute concentrations as well as the presence of other uncommon molecules are reflective of physiological conditions and can be utilized for disease diagnosis such as urinary tract infection (Simerville et al., 2005). However, urinary biomarkers can present in concentrations well below the limits of detection (LOD) of common diagnostic assays (Nimse et al., 2016). For example, the concentration of human growth hormone (hGH), a urinary biomarker, is ca. 100-fold below the immunoassays’ LOD (Fredolini et al., 2008). Urinary cell-free DNA can be utilized as a biomarker for cancer and infectious disease diagnostics (e.g., tuberculosis), but the dilute concentration and fragmented nature of the analyte impair the efficiency of extraction methods and consequently lower the diagnostic sensitivity (Oreskovic et al., 2019). The presence of high salts and interfering molecules (e.g., biotin) in urine also hinders the development and clinical implementation of urine-based diagnostic tests (Wong and Tse, 2009; Bowen et al., 2019).

Lateral flow assays (LFAs) are low-cost immunoassays for rapid biomarker detection, which have been widely used in medicine, environmental health, and quality control (Koczula and Gallotta, 2016). However, the LFAs’ detection limits are higher than the corresponding laboratory-based assays, so their sensitivities are significantly impacted by the low analyte concentrations and interferences (Moghadam et al., 2015; Zhang et al., 2020). For example, the Alere Determine TB-LAM Ag, a LFA for detecting urinary tuberculosis (TB) antigen lipoarabinomannan (LAM), has been proven to be highly specific and exhibits potential to be a high-impact point-of-care test (Peter et al., 2015). However, the estimated assay sensitivities are ∼18% for HIV-negative and ∼42% for HIV-positive individuals, caused by the low analyte concentration (Bulterys et al., 2019). The LAM concentration in urine for TB-positive patients can be as low as 14 pg/ml, which is significantly lower than the assay LOD, 500–1,000 pg/ml (Bulterys et al., 2019; García et al., 2019). Thus, the test cannot be utilized for general TB screening (World Health Organization, 2020).

Techniques have been developed to improve the sensitivities of LFAs, including kinetics and transport control (Yang et al., 2013; Rivas et al., 2014; Tang et al., 2017; Ishii et al., 2018), biochemical signal amplification (Hu et al., 2013; Parolo et al., 2013; Panferov et al., 2016), improved labeling (Choi et al., 2010), and sample enrichment. Sample enrichment techniques include centrifugal filtration (Corstjens et al., 2015), immunomagnetic separation (Panferov et al., 2017; Ben Aissa et al., 2021), electrophoretic and phasic separation (Wu et al., 2014; Chiu et al., 2015; Kim et al., 2017), isotachophoresis (Moghadam et al., 2015), dialysis (Tang et al., 2016), and test-zone pre-enrichment (Zhang et al., 2020). These systems are not suitable for low-resource, point-of-care settings as they require expensive reagents, equipment, and/or complex procedures. On the other hand, paper-based methods using dialysis or test-zone pre-enrichment lead to suboptimal enrichment and have limited processing capacity (Tang et al., 2016; Zhang et al., 2020). Therefore, there is a need for simple, versatile and effective approaches that enrich analytes to improve the LFA sensitivity.

To enable sensitive and rapid urinary biomarker detection *via* LFAs, this work presents the osmotic processor—a device that concentrates analytes via osmosis. The process spontaneously removes water molecules from the urine specimen while retaining the target analyte. The device includes a urine specimen compartment and a polymer compartment, which are separated by a semipermeable membrane. The polymer solution induces a strong osmotic pressure difference across the semipermeable membrane, which drives water molecules in the urine specimen across the membrane into the polymer solution (Nelson, 2017). Additionally, the membrane’s molecular weight cutoff (MWCO) is significantly smaller than that of the target analyte, allowing small ions and solutes that may interfere with the assay to be removed. Concentrating urinary analytes *via* osmosis has been demonstrated by McFarland, using cellulose acetate membrane and sucrose (polymer) to concentrate the analytes 5-fold for gel electrophoresis (McFarlane, 1964).

Compared to existing enrichment approaches, the osmotic processor demonstrates the potential to streamline its interface with existing LFAs, its simplicity with a spontaneous process, its flexibility to process a large specimen volume, and its capability to simultaneously recondition the concentrated specimen (remove inhibitory factors) for optimal assay performance. In this work the device was utilized to improve the detection limits of commercially available LFAs using human chorionic gonadotropin (hCG) and SARS-CoV-2 nucleocapsid (N) protein as model analytes. The osmotic processor has demonstrated ca. 100-fold concentration from a 10 ml sample for both analytes.

## 2 Materials and Methods

### 2.1 Materials

Polyethylene glycol 1,500/PEG 1500 (Sigma-Aldrich, St. Louis, MO, United States)**,** Polyethylene glycol 4,000/PEG 4000 (Sigma-Aldrich, St. Louis, MO, United States), Polyethylene glycol 35,000/PEG 35000 (Sigma-Aldrich, St. Louis, MO, United States), Poly (sodium 4-styrenesulfonate)/PSS (Sigma-Aldrich, St. Louis, MO, United States), Pectin (Spectrum Chemical Mfg. Corp, Gardena, CA, United States), Poly (acrylic acid sodium salt)/PAA (Sigma-Aldrich, St. Louis, MO, United States), Polyethyleneimine/PEI (Sigma-Aldrich, St. Louis, MO, United States), Spectra/Por 1 Dry Standard Grade Regenerated Cellulose (RC) Dialysis Tubing (Repligen, Waltham, MA, United States, 32 mm flat width, 6 kD, 1 m), SnakeSkin Dialysis Tubing (Thermo Fisher Scientific, Waltham, MA, United States, 3.5 K, 35 mm dry inner diameter, 35 feet), Original Prusa i3 MK3S + Printer (Prusa Research 3D, Prague, Czech Republic), Polylactic Acid (PLA) 1.75 mm Filament (Hatchbox3D, Pomona, CA, United States), Ammonium bicarbonate (ThermoFisher Scientific, Waltham, MA, United States, 99% for analysis), Chorionic gonadotropin human (Sigma-Aldrich, St. Louis, MO, United States, 5000 IU lyophilized powder), AimStep Pregnancy Urine Cassette Test (Germaine Laboratories, Inc. San Antonio, TX, United States of America), Nucleocapsid Protein 95% COVID-19 (ACROBiosystems, Newark, DE, United States), Quidel QuickVue at-Home OTC COVID-19 Test (Quidel Corporation, San Diego, CA, United States), Urea (Bio Rad Lab, Hercules, CA, United States, Pkg of 1, 250 g), TCEP (Promega Corporation, Madison, WI, 15 mg), Iodoacetamide (Thermo Fisher Scientific, Waltham, MA, United States), Dithiothreitol (Bio Rad Lab, Hercules, CA, United States, 1 g), Trypsin (Sigma-Aldrich, St. Louis, MO, United States, 1x Gamma-Irradiated 0.25% Porcine Trypsin 1:250 in HBSS w/0.1% EDTA-NA2 w/o CA and MG), formic acid (Fisher Scientific, Waltham, MA, United States, 0.1% in water, Optima LC/MS, Solvent Blends), Orbitrap Exploris 480 mass spectrophotometer (Thermo Fisher Scientific, Waltham, MA, United States), EASYnLC 1200 UPLC system (Thermo Fisher Scientific, Waltham, MA, United States), Analytical column (New Objective, Inc., Woburn, MA, ID 75 μm), Integrafrit trap column (New Objective, Inc., Woburn, MA, ID 100 µm), ReprosilPur C18AQ 5 µm beads (Dr. Maisch, Tubigen, Germany), formic acid (Fisher Scientific, Waltham, MA, United States, 0.1% in acetonitrile, Optima LC/MS, Solvent Blends), Water (Fisher Scientific, Waltham, MA, United States, Optima™ LC/MS Grade)

### 2.2 Methods

#### 2.2.1 Water Soluble Polymers for Osmosis

Various water-soluble polymers were utilized to evaluate the rate of water movement across the membrane, driven by osmosis. The evaluation included polymers with different compositions, molecular weights, and charge properties. The polymer characteristics and the solution concentrations are summarized in Table 1.

**TABLE 1:** Polymers prepared at maximum mass concentration.

Polymer	Maximum mass Concentration (g/ml)	Molecular weight (daltons)	Charge
PEG 1500	2	1,500	neutral
PEG 4000	1.5	4,000	neutral
PEG 35000	0.8	35,000	neutral
PSS	0.6	1,000,000	negative
Pectin	0.25	Unknown	neutral
PAA	1	5,100	negative
PEI	1	25,000	positive

In this study, 10 ml deionized water was loaded in the Spectra/Por 1 dialysis tubing with 32 mm flat width and 6–8 kDa MWCO. Then, the sealed dialysis bags were immersed in 80 ml of a polymer solution, listed in Table 1. After 30 min, the dialysis bag was removed from the polymer solution, and then briefly rinsed with DI water to remove excess polymer. The remaining water in the dialysis bag was transferred to a graduated cylinder for volume measurement. The volume difference between the initial 10 ml solution and remaining liquid was the total water removed, which was divided by the processing time (0.5 h) to estimate the water removal rate.

#### 2.2.2 Effect of Polymer Molecular Weight on Osmosis

To evaluate the effect of polymer molecular weight on the rate of water transport across the membrane, the study utilized polyethylene glycol (PEG) at three different molecular weights, 1.5, 4, and 35 kDa. All polymer solutions were prepared by pre-dissolving the polymers in deionized water at 0.8 g/ml to drive the osmosis. For the evaluation, 10 ml deionized water was loaded in the Spectra/Por 1 dialysis tubing with 32 mm flat width and 6–8 kDa MWCO. Then, the sealed dialysis bags were immersed in 80 ml of the PEG solution. After 30 min, the dialysis bag was removed from the polymer solution, and then briefly rinsed with DI water to remove excess polymer. The remaining water in the dialysis bag was transferred to a graduated cylinder for volume measurement. The volume difference between the initial 10 ml solution and remaining liquid was the total water removed, which was divided by the processing time (0.5 h) to estimate the water removal rate.

#### 2.2.3 Effect of Polymer Solution Mass Concentration on Osmosis

To evaluate the effect of polymer solution mass concentration on the rate of water transport across the membrane, the polymer solutions were prepared using PEG 1500 (1.5 kDa PEG) to drive the osmosis. Specifically, PEG 1500 was dissolved in deionized water at concentrations of 0.125, 0.25, 0.5, 1.0, or 2.0 g/ml. For the evaluation, 10 ml deionized water was loaded in the Spectra/Por 1 dialysis tubing with 32 mm flat width and 6–8 kDa MWCO. Then, the sealed dialysis bags were immersed in 80 ml of the PEG solution. After 30 min, the dialysis bag was removed from the polymer solution, and then briefly rinsed with DI water to remove excess polymer. The remaining water in the dialysis bag was transferred to a graduated cylinder for volume measurement. The volume difference between the initial 10 ml solution and remaining liquid was the total water removed, which was divided by the processing time (0.5 h) to estimate the water removal rate.

#### 2.2.4 Osmotic Processor Fabrication and Assembly

**SCHEME 1:**
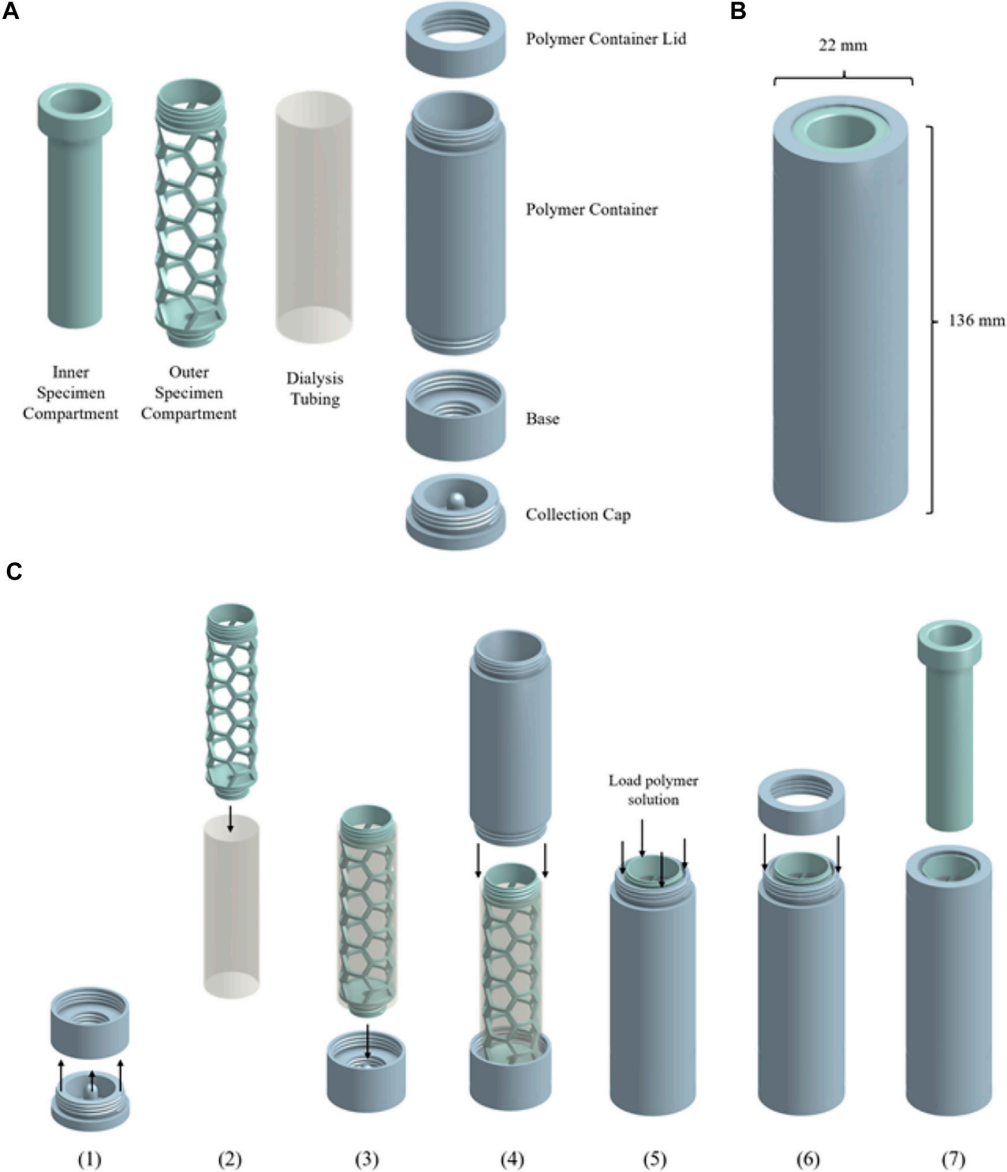
**(A)** Components of the osmotic processor: urine compartment, dialysis tubing, polymer container lid, polymer container, base and collection cap. **(B)** Dimensions of the osmotic processor. **(C)** Assembly workflow of the osmotic processor. (Created with Onshape).

Scheme 1 shows the device components, dimension, and assembly workflow. The urine compartment, polymer container lid, polymer container, base, and collection cap components of the osmotic processor (Scheme 1A) were fabricated using the fused filament fabrication (FFF) 3D printing method on the Original Prusa i3 MK3S + printer (Prusa Research 3D, Prague, Czech Republic) with 1.75 mm PLA filament. The height and diameter of the assembled device were 136 and 22 mm, respectively (Scheme 1B). Scheme 1C illustrates the assembly workflow. 1) The sample collection cap was secured to the base of the device. 2) The outer specimen compartment was inserted into 150 mm of SnakeSkin dialysis tubing with 35 mm inner diameter to hold the structure of the membrane. 3) The outer urine compartment was screwed onto the base to secure the bottom end of the dialysis tubing. 4) The polymer container was screwed onto the base. 5) The polymer container was filled with 50 ml of polymer solution. 6) The polymer container was sealed with the polymer container lid. 7) The assembled device is ready for specimen to be added. See Scheme 3 for the osmotic processing workflow.

#### 2.2.5 Concentrating Human Chorionic Gonadotropin (hCG) Hormone for Improved Lateral Flow Assay Detection Limit

hCG solutions were processed using the osmotic processor, and then the concentrated specimens were assayed using the AimStep pregnancy test, a LFA. The osmotic processor utilized 50 ml of PEG 1500 at 2 g/ml and the 6–8 kDa Spectra/Por 1 dialysis tubing. Specimens were prepared by diluting hCG in deionized water. 10 ml of the 0.02 μg/ml hCG solution was processed for 45 min, and the resulting 100 µl sample was assayed using the AimStep pregnancy test by following the vendor’s protocol. 0 μg/ml hCG solution was also processed and assayed as a negative control. For reference, the stock solutions with 0, 0.02, and 2 μg/ml hCG wereassayed using the AimStep pregnancy test. The results were recorded by capturing the images of the assays, using an Epson Perfection v39 photo scanner. The test band intensity of the hCG LFA was analyzed with ImageJ (Schneider et al., 2012), an image-processing software, to produce semi-quantitative comparison between the specimens before and after the osmosis. The signal was measured in arbitrary units by inverting the image color, selecting an area on the test band, and measuring the raw integrated pixel density of the selected area. For the semi-quantitative comparison, the test line signals were further processed by normalizing against the background noise, signals from a no color membrane area away from the test and control bands.

#### 2.2.6 Concentrating SARS-CoV-2 Nucleocapsid (N) Protein for Improved Lateral Flow Assay Detection Limit

Solutions of SARS-CoV-2 N protein were processed using the osmotic processor, and then assayed with the QuickVue test, a LFA. The osmotic processor utilized 50 ml of PEG 35000 at 0.8 g/ml and the 3.5 kDa SnakeSkin dialysis tubing. Specimens were prepared by diluting the SARS-CoV-2 N protein in the 50 mM ammonium bicarbonate buffer. 10 ml of the 0.04 ng/mL N protein solution was concentrated by the osmotic processor for 1.5 h, and the resulting 100 µl sample was assayed using the QuickVue test. 0 ng/ml N protein solution was also processed and assayed as a negative control. For reference, the stock solutions with 0, 0.04, and 4 ng/mL N protein were assayed using the QuickVue test. The results were recorded by capturing the assay membrane images, using an Epson Perfection v39 photo scanner. The test band intensity of the SARS-CoV-2 LFA was analyzed with ImageJ (Schneider et al., 2012), an image-processing software, to produce semi-quantitative comparison between the specimens before and after the osmosis process. The signal was measured in arbitrary units by inverting the image color, selecting an area on the test band, and measuring the raw integrated pixel density of the selected area. For the semi-quantitative comparison, the test line signals were further processed by normalizing against the background noise, signals from a no color membrane area away from the test and control bands.

#### 2.2.7 Mass Spectrometry Assays for Quantitating Analyte Enrichment via the Osmosis Processor

The analyte enrichment was also characterized via mass spectrometry using SARS-CoV-2 N protein as the model analyte. To accommodate the mass spectrometry LOD, which is significantly higher than LFA, the evaluation utilized specimens with 4 μg/ml N protein. The aforementioned approach (2.2.6) was employed to concentrate SARS-CoV-2 N protein via the osmotic processor. A calibration was generated by performing mass spec analysis using 100 µL of standard solutions with 0, 0.08, 0.4, 2, 10 and 40 μg/ml N protein.

Specimen preparation for mass spectrometry followed a published protocol (Beynon et al., 1989). In brief, reduction was carried out by incubating 100 µl of each sample with 2.5 µl of 200 mM TCEP at 37°C for 1 h, followed by alkylation with 20 µl of 200 mM iodoacetamide at room temperature in the dark. Excess iodoacetamide was quenched by incubation of the sample with 20 µl 200 mM DTT at room temperature. The samples were diluted by adding 700 µl of 50 mM ammonium bicarbonate, after which tryptic digestion was carried out at 37°C overnight. Prior to liquid chromatography/mass spectrometry (LC/MS) analysis, all samples were dried down and resuspended in 0.1% formic acid in water.

All samples were analyzed on an Orbitrap Exploris 480 mass spectrophotometer equipped with an EASYnLC 1200 UPLC system and an in-house developed nano spray ionization source. Samples (5 μl at various concentrations) were loaded from the autosampler onto a 100 μm ID Integrafrit trap packed with Reprosil-Pur C18-AQ 120 Å 5 µm material to a bed length of 2.5 cm with a volume of 18 μl at a flow rate of 2.5 μl/min. After loading and desalting with 0.1% formic acid in water, the trap was brought in-line with a pulled fused-silica capillary tip (75-μm i. d.) packed with 35 cm of Reprosil-Pur C18-AQ 120 Å 5 µm. Peptides were separated using a linear gradient, from 6–45% solvent B (LCMS grade 0.1% formic acid, 80% acetonitrile in water) in 60 min at a flow rate of 300 nL/min. Peptides were detected using a targeted Parallel Reaction Monitoring (PRM) method. After the survey scan, targeted MS/MS was performed based on the inclusion list of 23 precursors (m/z, charge state) generated by Skyline (Pino et al., 2020). Precursors were isolated in the quadrupole with an isolation width of 2 m/z. Higher-energy collisional dissociation (HCD) fragmentation was applied with a normalized collision energy of 30% and resulting fragments were detected in the Orbitrap mass analyzer at 15 k resolution (at 200 m/z) with a 300% ion count (AGC) target and a maximum injection time of 22 ms. The loop count was set to ‘All’, to generate 23 fragment ion spectra per MS1 scan.

Data processing and analysis were performed using Skyline (Pino et al., 2020). The raw data were imported and R. ITFGGPSDSTGSNQNGER.S [15, 32], the peptide sequence, was selected for analysis due to its high abundance. The peak area under the intensity vs. retention time curve of the selected sequence was calculated by the Skyline software and correlated to the N protein standards’ concentration as the calibration. Then, the peak areas of the unprocessed and processed N protein samples were used to estimate the specimen concentration via the calibration.

## 3 Results and Discussion

### 3.1 Osmosis Driven by the Polymer Solutions

**SCHEME 2:**
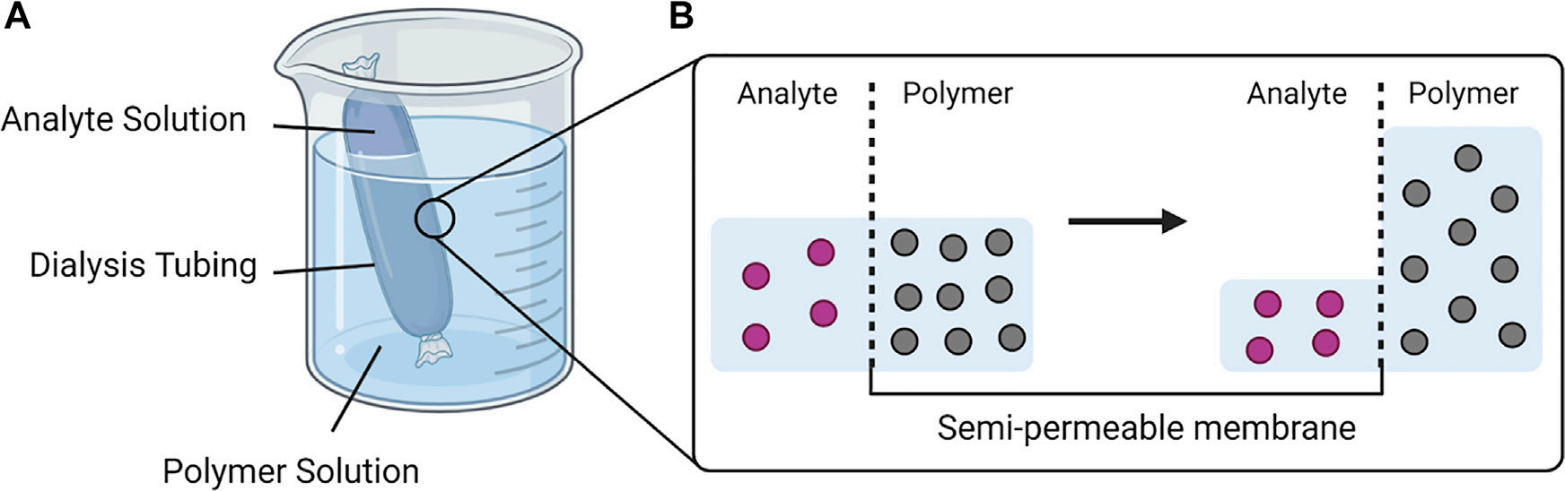
The process of osmosis applied in the urine specimen processor. **(A)** The analyte solution in a sealed dialysis tubing is immersed in a highly concentrated polymer solution. **(B)** The osmotic pressure difference drives the water molecules from the analyte solution across the semipermeable membrane to the polymer solution until equilibrium is reached. (Created with BioRender).

To concentrate the urinary biomarkers, we have designed and fabricated devices that employ osmosis to remove water molecules from the specimens, illustrated in Scheme 2. The beaker contains an aqueous polymer solution and a dialysis bag with the specimen inside (e.g., urine). The osmosis occurs when the dialysis bag is placed in the polymer solution due to the osmotic pressure generated by the polymer solution. The membrane MWCO is small enough to retain the analyte while allowing the transport of water and other smaller molecules. The rate of water transport across the semipermeable membrane with regard to osmotic pressure (π) can be described by the following equation (Lucke et al., 1931):

$$\frac{dV}{dt}=k\times S\times \left(\pi -P_{ex}\right)$$ (1)

where dV/dt is the rate of change of volume, k is the membrane permeability, S is the surface area of the membrane and P_ex_ is the surrounding pressure. The osmotic pressure (π) produced by non-ideal polymer solutions can be described using the Flory-Huggins equation (Flory, 1942; Huggins, 1942):

$$\pi =c\times R\times T\left(M^{-1}+A_{2}c+A_{3}c^{2}+\cdots \right)$$ (2)

where c is the mass concentration of the polymer solution, R is the gas constant, T is the system temperature, M is the polymer molecular weight, and A_2_ and A_3_ are osmotic virial constants that describe the polymer-solvent interaction. The van’t Hoff theory is commonly applied to describe principle of osmosis in “ideal” solutions by assuming the solute and solvent particles are of similar sizes and occupy similar volumes (Kendall, 1921). However, the equation cannot be used here because the polymer (solute) is much larger in size and occupies more volume than the water molecules (solvent). Therefore, the Flory-Huggins equation is applied here to reflect the contribution of non-ideality to the solution osmotic pressure due to the difference in solute and solvent molecular sizes.

**FIGURE 1:**
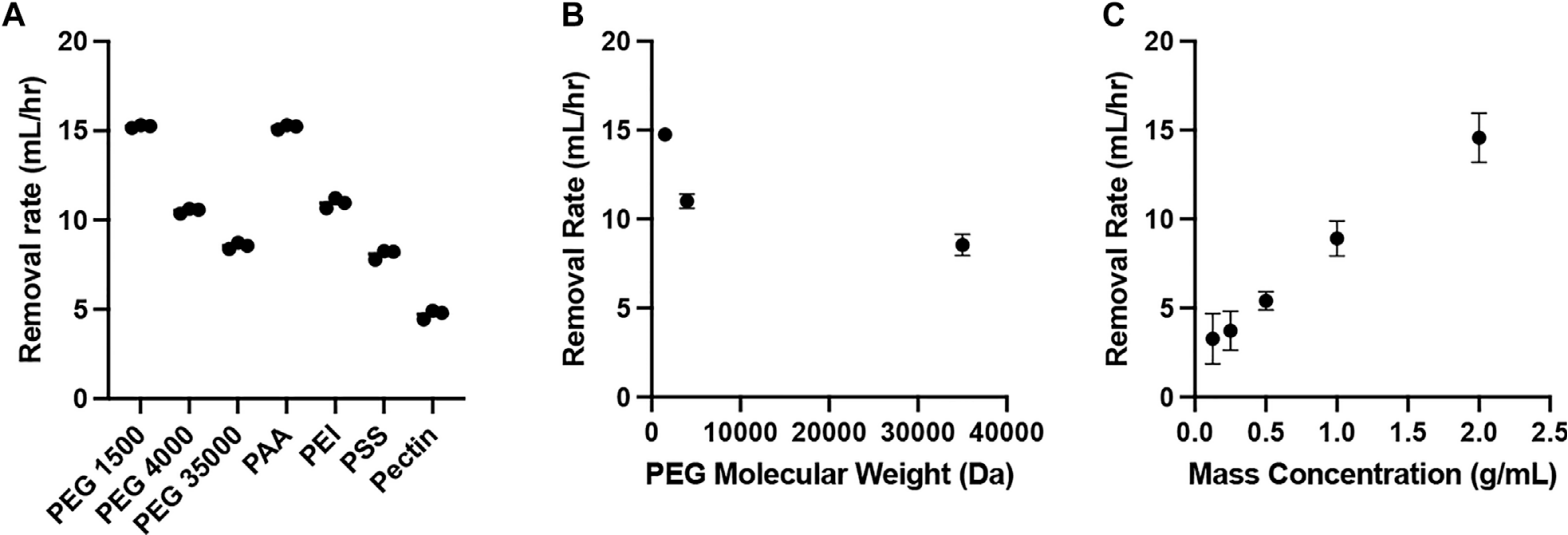
Water removal rate (*n* = 3). **(A)** Various polymers, including PEG 1500, PEG 4000, PEG 35000, PAA, PEI, PSS, and Pectin; **(B)** Polymer solutions (0.8 g/ml) using PEG with 1500, 4000 and 35000 Da molecular weights; **(C)** Polymer solutions using PEG 1500 with mass concentrations of 0.125, 0.25, 0.5, 1.0 and 2.0 g/ml.

The water removal *via* osmosis was demonstrated using various water soluble polymers (Table 1), including poly (ethylene glycol)/PEG, poly (acrylic acid)/PAAc, polyethylenimine/PEI, poly (sodium 4-styrenesulfonate)/PSS, and pectin. The evaluation utilized the apparatus illustrated in Scheme 2. The rate of water removed from the dialysis bag varied from ca. 5–15 ml/h, and was highest for PEG 1500, followed by PAA, PEI, PEG 4000, PEG 35000, PSS and Pectin (Figure 1A). Because lower molecular weight leads to higher water solubility, PEG 1500 was prepared at a higher mass concentration (2 mg/ml), resulting in a higher osmotic pressure (Eq. 2) across the dialysis membrane to drive 15.2 ± 0.1 ml/h water removal. The removal rates were 10.5 ± 0.1 and 8.6 ± 0.2 ml/h for PEG 4000 and PEG 35000 respectively. The stronger polymer-water interaction via charge and the low molecular weight, 5.1 kDa, allowed the PAA solution to be prepared at a higher mass concentration, 1 g/ml, resulting in 15.2 ± 0.1 ml/h of water removed. The removal rates were 10.9 ± 0.3 and 8.1 ± 0.3 ml/h for PEI and PSS, respectively. The slower water removal associated with PEI and PSS is likely caused by lower osmotic pressure, which is a function of mass concentration (Eq. 2). While PEI and PSS are both charged polymers, higher molecular weights (25 kDa for PEI and 1,000 kDa for PSS) limit the solubilities, which lead to lower osmotic pressures. Pectin is not charged and likely has high molecular weight given the low maximum mass concentration, with 4.7 ± 0.3 ml/h water removed.

According to the Flory-Huggins equation (Eq. 2), the osmotic pressure is a function of polymer molecular weight. Therefore, we further evaluate the osmosis using polymer solutions prepared by PEG with different molecular weights. All polymer solutions had the same mass concentration, 0.8 g/ml. The results are summarized in Figure 1B. As the molecular weight of PEG increases from 1.5 to 35 kDa, the rate of water removal from the dialysis tubing decreased from 14.8 ± 0.4 to 8.6 ± 0.6 ml/h. The observed phenomenon aligns with the inverse relationship between polymer molecular weight and osmotic pressure (Eq. 2). Higher polymer molecular weight results in lower osmotic pressure, which slows down the change in water volume over time in the dialysis tubing (Eq. 1).

We also evaluated the impact of polymer solution mass concentration for osmosis. According to the Flory-Huggins equation (Eq. 2), higher mass concentration leads to stronger osmotic pressure. PEG 1500 solutions at concentrations of 0.125, 0.25, 0.5, 1, and 2 g/ml were prepared for the evaluation. The results are summarized in Figure 1C. As the polymer solution concentration increased from 0.125 to 2 g/ml, the rate of water removal from the dialysis tubing increased from 3.3 ± 1.4 to 14.6 ± 1.4 ml/h. The increase in polymer mass concentration results in a proportional increase of osmotic pressure, increasing the rate of water transport across the membrane (Eqs 1 and 2).

### 3.2 Osmotic Processor Design Specifications and Workflow

**SCHEME 3:**
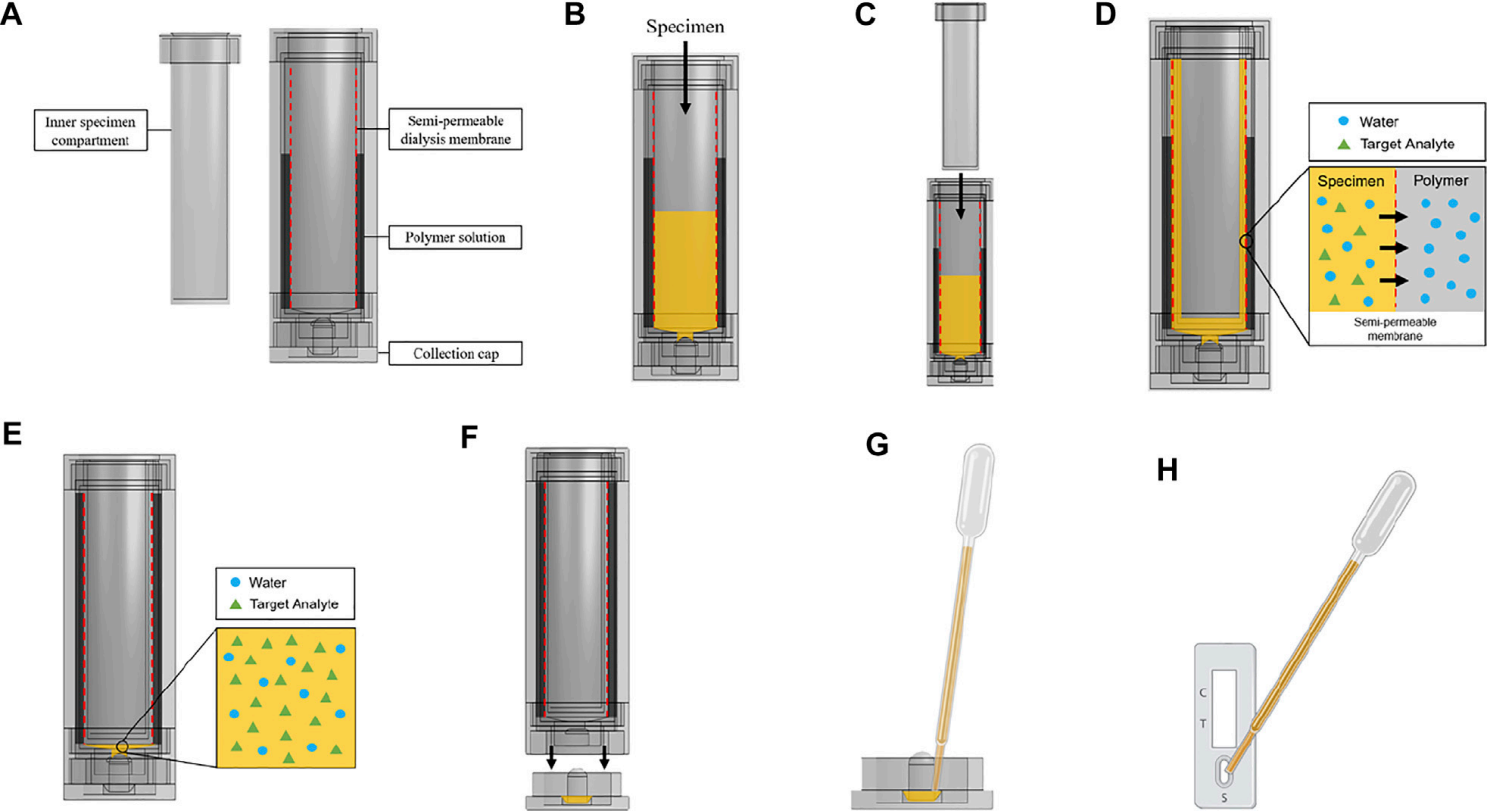
Osmotic specimen processing device design and workflow. **(A)** Device components. **(B)** Load sample into urine compartment. **(C)** Insert inner component of urine compartment to create thin specimen layer against dialysis membrane. **(D)** Water from the analyte solution is drawn out by the polymer solution through the process of osmosis. The target analyte is retained by a semipermeable membrane. **(E)** The processed specimen is collected at the bottom of the urine compartment with a specific volume cutoff. **(F)** The specimen flows from the urine compartment to the collection cap when the collection cap is unscrewed. **(G)** The processed specimen is pipetted from the collection cap. **(H)** The processed specimen is deposited onto the lateral flow test. (Created with OnShape and BioRender).

Based on our evaluation (Figures 1A–C), the Flory-Huggins equation (Eq. 2), and reagent availability, PEG was chosen for the osmotic processor. Assembled components of the specimen processor are shown in Scheme 3A. The workflow starts from adding the specimen to the device (Scheme 3B). Then, the inner component is insert to the urine compartment to create a thin specimen layer (Scheme 3C). To maximize membrane surface area (Eq. 1), the interface between the specimen and the polymer solution, the urine specimen compartment is designed to create a thin cylindrical layer that presses against the semipermeable membrane in contact with the surrounding polymer solution. The 3.5 kDa MWCO, 35 mm dry inner diameter SnakeSkin dialysis tubing was selected to effectively retain the targeted analytes such as hCG (∼36 kDa) and the SARS-CoV-2 N protein (∼114 kDa). The polymer solution creates a pressure difference across the membrane, driving water transport from the urine compartment to the polymer container (Scheme 3D). To prevent the over-concentration and sample dry-out, a small collection cup (Scheme 3E) that has no contact with the membrane was designed at the bottom of the specimen compartment. Therefore, the final volume of the concentrated specimen is fixed at 100 µl. To collect the processed sample, the collection cup can be detached from the bottom of the device (Scheme 3F), and the sample can be deposited onto the lateral assay by pipetting (Schemes 3G,H).

### 3.3 Concentrating Human Chorionic Gonadotropin Hormone to Improve Lateral Flow Assay Detection Limit

**FIGURE 2:**
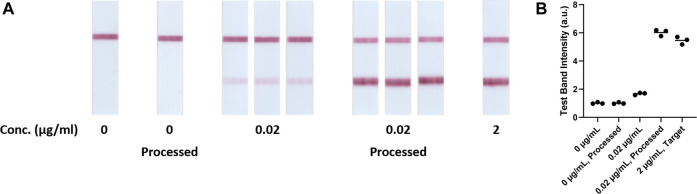
**(A)** 0 μg/mL, 0 μg/mL processed, 0.02 μg/mL, 0.02 μg/mL processed and 2 μg/mL target hCG samples on AimStep hCG Pregnancy Lateral Flow Cassette (*n* = 3). **(B)** Test band intensities of blank, unprocessed, target and processed hCG LFA tests (*n* = 3).

To demonstrate the improved LFA detection limit, hCG hormone and AimStep^®^ Pregnancy (a LFA) were used as a model system. The qualitative results were recorded by capturing the images of the assays (Figure 2A), which were analyzed by quantitating the test line intensities as the assay signals (Figure 2B). In this evaluation, assays with 0, 0.02, and 2 μg/ml hCG solutions were used as references. The device was utilized to process a 10 ml solution with 0 and 0.02 μg/ml hCG. After 45 min, the resulting 100 µl concentrated specimens were assayed using AimStep^®^ Pregnancy. Assays with both 0 μg/ml and processed 0 μg/ml hCG solutions resulted in only a visible control line, which were correctly determined as negatives. Solutions with 0.02 and 2 μg/ml hCG resulted in both visible control and test lines, which were classified as positives. The 0.02 μg/ml hCG solutions led to faint test lines, and the test line intensities of the assays with 2 μg/ml hCG specimens were significantly higher (Figure 2A). After the osmotic processing, the 0.02 μg/ml hCG specimens resulted in much stronger test line intensities, which were similar to the 2 μg/ml hCG assays. Assay signals, generated via ImageJ analysis (Figure 2B), were 1.01 ± 0.06, 1.68 ± 0.08, and 5.46 ± 0.27 for specimens with 0, 0.02, and 2 μg/ml hCG respectively. After the osmosis, the assay signals of the 0 μg/ml hCG controls was 1.01 ± 0.06. The assay signals for the processed 0.02 μg/ml specimens increased to 6.02 ± 0.23, which were almost the same as the assays with 2 μg/ml hCG, indicating a ca. 100-fold concentration.

### 3.4 Concentrating SARS-CoV-2 Nucleocapsid Protein to Improve Lateral Flow Assay Detection Limit

**FIGURE 3:**
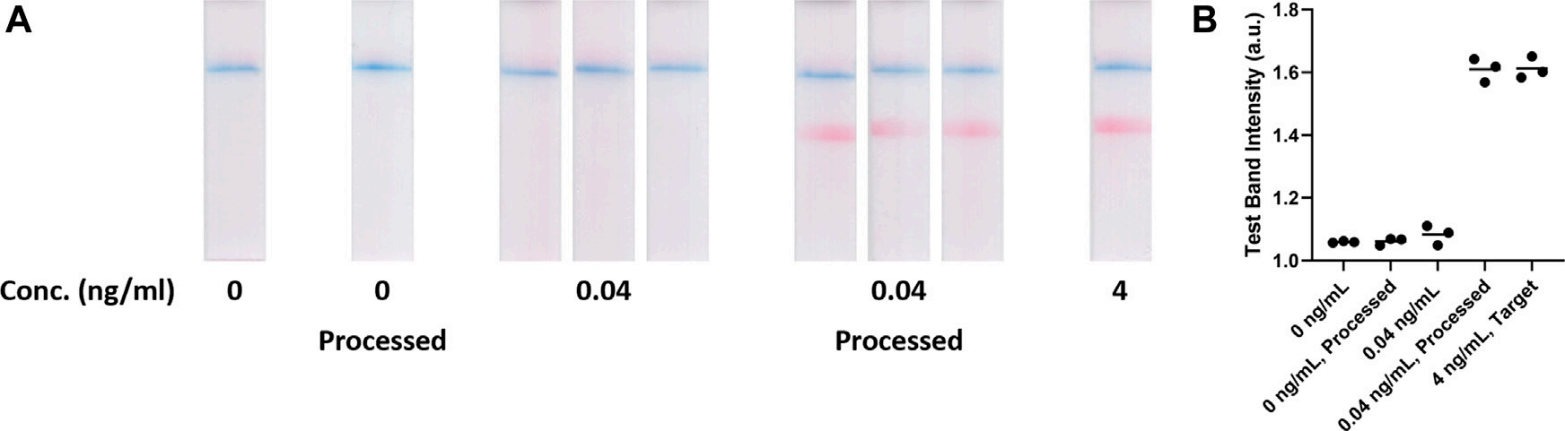
**(A)** 0 ng/mL, 0 ng/mL processed, 0.04 ng/mL, 0.04 ng/mL processed and 4 ng/mL target N protein samples on Quidel QuickVue SARS-CoV-2 Lateral Flow Assays (*n* = 3). **(B)** Test band intensities of blank, unprocessed, target and processed SARS-CoV-2 LFA tests (*n* = 3).

To further demonstrate the device versatility for improving LFA detection limit, SARS-CoV-2 nucleocapsid (N) protein and the QuickVue test (a LFA) were used as a model system. The evaluation was carried out using an approach similar to the aforementioned hCG model system. The qualitative results were recorded by capturing images of the assays (Figure 3A), which were analyzed by quantitating the test line intensities as the assay signals (Figure 3B). In this evaluation, assays with 0, 0.04, and 4 ng/ml N protein solutions were used as references. Figure 3A shows the assays with 0 ng/ml and processed 0 ng/ml N protein resulted in only a visible control line, which were determined as negatives. Specimens with 0.04 ng/ml N protein resulted in only a visible control line but specimens with 4 ng/ml N protein resulted in both visible control and test lines. The results indicated that the specimens with 0.04 ng/ml N protein were below the assay LOD. After the osmotic processing, the 0.04 ng/ml N protein specimens resulted in much stronger test lines, which were similar to the 4 ng/ml N protein assays. Assay signals, generated *via* ImageJ analysis (Figure 3B), were 1.06 ± 0.01, 1.08 ± 0.03, and 1.61 ± 0.03 for specimens with 0, 0.04, and 4 ng/ml N protein, respectively. After the osmosis, the assay signal for the 0 ng/ml N protein solution was 1.06 ± 0.01. The assay signal for the processed 0.04 ng/ml N protein specimens increased to 1.61 ± 0.04, which was almost the same as the assays with 4 ng/ml N protein, indicating a ca. 100-fold concentration.

**FIGURE 4:**
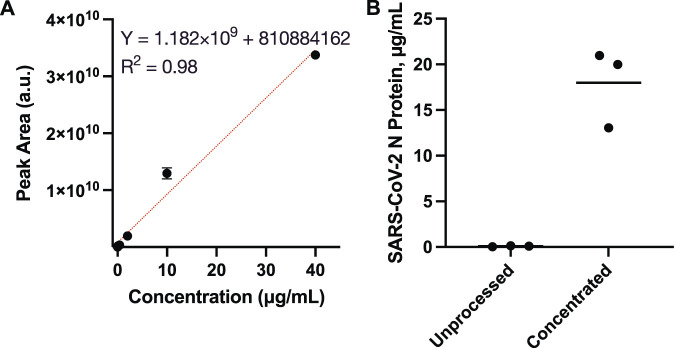
Mass spectrometry analysis for quantitating SARS-CoV-2 N protein concentration by measuring the area under the ionization intensity vs. retention time peaks of the peptide sequence R.ITFGGPSDSTGSNQNGER.S [15, 32]. **(A)** Calibration using standards, 0, 0.08, 0.4, 2, 10 and 40 μg/ml SARS-CoV-2 N protein; **(B)** Specimens’ SARS-CoV-2 N protein concentrations before and after the osmosis. The solution average concentration was 0.09 ± 0.06 μg/ml prior to the process. Then, the concentration increased to 18.0 ± 4.3 μg/ml N protein, nearly 200-fold, after the enrichment.

Mass spectrometry was utilized to quantitate SARS-CoV-2 N protein concentrations, which were used to estimate the enrichment factor via the osmotic processor. The enrichment factors are the concentration ratios of the processed specimens over the unprocessed solutions. To accommodate the mass spectrometry dynamic range, the evaluation utilized specimens with 4 μg/ml SARS-CoV-2 N protein for the concentration process. Skyline, a software for targeted proteomics data analysis, was utilized to measure the area under the ionization intensity vs. retention time peaks of the peptide sequence R. ITFGGPSDSTGSNQNGER.S [15, 32] for the standards (0, 0.08, 0.4, 2, 10 and 40 μg/ml SARS-CoV-2 N protein) as well as the specimens before and after the concentration process (Supplementary Materials, the peak near 26 min in Supplementary Figure S1). Because of the trypsin digestion, the specimens were diluted 10-fold prior to the mass spectrometry. The standards were utilized to generate a calibration (Figure 4A), which was used to determine the specimen concentrations. Prior to osmosis, the specimens’ average N protein concentration was 0.09 ± 0.06 μg/ml (Figure 4B). After the concentration process, the specimens contained 18.0 ± 4.3 μg/ml N protein. The mass spectrometry analysis indicated that the osmotic processor concentrated the specimen nearly 200-fold. The specimen concentrations were lower than the theoretical values, which might be caused by the loss during the sample preparation.

In this study, an osmotic processor was developed to spontaneously concentrate analytes for improving the LOD of existing LFAs. The processor employed solutions with water-soluble polymers to create osmotic pressure difference across the membrane to drive the water transport, removing water from the specimen to concentrate analytes. Several polymers were evaluated and have demonstrated the capability to induce osmosis. However, the rate of water transport varies because of the polymer properties (e.g., molecular weight, charge properties). The systematic evaluations showed that faster water transport can be induced using PEG with lower molecular weights and the polymer solutions with higher mass concentration. According to the Flory-Huggins principle (Eq. 2), these PEG solutions resulted in higher osmotic pressures, which drove faster water transport *via* osmosis (Eq. 1). Therefore, PEG solutions were incorporated into the osmotic processor. To further increase the water transport rate, the specimen compartment of the processor utilizes an insert that results in a thin layer specimen solution on the membrane to maximize the contact surface area. The osmotic processor was utilized to concentrate analytes for more sensitive biomarker detections via LFAs, using hCG and SARS-CoV-2 N protein as the model analytes. After the osmosis, LFAs showed strong signals for the solutions with low analyte concentration. The analyses for the LFA membranes suggest the analyte concentrations were increased nearly 100-fold for both model analytes. Additional quantitative analyses via mass spectrometry showed the concentration of SARS-CoV-2 N protein increases ca. 200-fold. Table 2 summarizes various analyte concentration approaches, which were utilized for proteins as well as nucleic acids. These approaches concentrated analytes from few to 400-fold. Compared to the existing approaches, the osmotic processor can achieve similar enrichment and can concentrate analyte spontaneously. The osmotic processor can be utilized in conjunction with LFAs to improve biomarker detection, which can potentially increase the assay sensitivity.

**TABLE 2:** Analyte enrichment by various concentration techniques.

Method	Sample type	Biomarker	Enrichment fold (ca.)
Osmotic Processor	Ammonium bicarbonate buffer	Human chorionic gonadotropin (hCG); SARS-CoV-2 N protein	100
Centrifugal Filtration (Corstjens et al., 2015)	Urine	Circulating cathodic antigen	100
Dialysis (Tang et al., 2016)	Water	Human Immunodeficiency Virus nucleic acid (HIV NA); myoglobin (MYO)	HIV NA: 4; MYO: 10
Electrophoresis (Wu et al., 2014)	Saline Sodium Citrate	DNA of H5 subtype of avian influenza virus	400
Aqueous two-phase system (Chiu et al., 2015)	PBS, FBS, synthetic urine	Transferrin	100
Isotachophoresis (Moghadam et al., 2015)	TE (*Glycine*, Bis-Tris, pH7.4)	Goat anti-rabbit IgG; Goat anti-mouse IgG	160–400
Test-zone pre-enrichment (Zhang et al., 2020)	human blood serum; PBS	miR-210 mimic; hCG	miR-210 mimic: 10–100; hCG: 10
Immunomagnetic separation (Panferov et al., 2017; Ben Aissa et al., 2021)	PBST; PBS	Potato virus X (PVX); 16S rRNA gene for *Myobacterium* (rGM)	PVX: 6; rGM: 10

## 4 Conclusion

We have fabricated an osmotic processor that can spontaneously concentrate specimens’ analyte to improve biomarker detections via LFA. Using hCG and SARS-CoV-2 N protein as model analytes, the osmotic processor has demonstrated the concentration process qualitatively and quantitatively. Specimens originally with analytes below the LFA LOD became detectable after the osmosis, indicating the osmotic processor concentrated the analyte to above the assay LOD. The quantitative analysis via mass spectrometry suggested ≥ 100-fold analyte concentration via the device. The device can potentially improve biomarker detection by interfacing with urine-based LFAs, a lot of which have problems with low-sensitivity. The analyte concentration via the osmotic processor is very comparable to other existing concentration approaches. The current design requires the transfer of enriched specimen to lateral flow test strips by manual pipetting. For further improvements in the point-of-care diagnostic workflow, the osmotic processor may be modified to seamlessly integrate with existing LFAs, where the concentrated specimen is directly released onto the sample pad of LFAs. Additionally, the device is easy to use, and does not depend on a power source, which can potentially enable many point-of-care tests (e.g., TB screening via urinary LAM) in low-resources settings.

## Data Availability

The original contributions presented in the study are included in the article/Supplementary Material, further inquiries can be directed to the corresponding author.
