# Supplementary material for: Osmotic Processor for Enabling Sensitive and Rapid Biomarker Detection via Lateral Flow Assays
Source: Front Bioeng Biotechnol. 2022 Jun 1;10:884271. doi: 10.3389/fbioe.2022.884271 (PMC9199386; doi:10.3389/fbioe.2022.884271)
Supplement: Supplementary file 1 [file DataSheet1.docx]

Osmotic Processor for Enabling Sensitive and Rapid Biomarker Detection via Lateral Flow Assays

Sheng-You Chen^1†^, Abe Y. Wu^2†^, Ruby Lunde^2†^, James J. Lai^2*^

^1^Department of Mechanical Engineering, University of Washington, Seattle, Washington, 18195

^2^Department of Bioengineering, University of Washington, Seattle, Washington, 18195

†These authors contributed equally to the work.

*** Correspondence:**James J. Lai
jilai@uw.edu

SFig. 1. Orbitrap mass spectrometry intensity vs. retention time chromatograms of peptide sequence R.ITFGGPSDSTGSNQNGER.S (precursor 912.4114++), the peak near 26 minutes, from (A) SARS-CoV-2 nucleocapsid protein standards—0, 0.08, 0.4, 2, 10, and 40 µg/mL; (B) before and after osmosis process for SARS-CoV-2 nucleocapsid protein specimens.
